# Inhibition of miR-206 Ameliorates Seizures and Cognitive Deficits via BDNF/TrkB Signaling in Pilocarpine-Induced TLE Mice

**DOI:** 10.3390/brainsci16101048

**Published:** 2026-09-29

**Authors:** Hua Zhang, Zhengxu Cai

**Affiliations:** Department of Research Center, Dalian Medical University, Dalian 116044, China; 19802460656@163.com

**Keywords:** epilepsy, cognitive function, miR-206

## Abstract

**Highlights:**

**What are the main findings?**

- Hippocampal miR-206 expression is significantly elevated in pilocarpine-induced chronic temporal lobe epilepsy (TLE) mice.
- Stereotactic hippocampal injection of miR-206 antagomir effectively downregulates endogenous miR-206 in the epileptic hippocampus. Inhibition of miR-206 prolongs seizure latency, reduces seizure frequency, and shortens seizure duration in TLE mice as verified by EEG monitoring. MiR-206 antagomir rescues spatial learning-memory impairments demonstrated by improved performance in the Morris water maze test. Suppressing miR-206 alleviates hippocampal CA3 neuronal loss and preserves neuronal integrity based on HE and Nissl-staining observations. MiR-206 inhibition upregulates BDNF/TrkB as well as synaptic-plasticity-associated proteins SYP and PSD95 in the hippocampus of TLE mice. Pharmacological blockade of TrkB by ANA-12 reverses the anti-seizure, cognitive-improving, and neuroprotective effects of miR-206 antagomir.

**What are the implications of the main findings?**

- The protective effects elicited by miR-206 inhibition are functionally dependent on intact activation of the hippocampal BDNF/TrkB signaling pathway in TLE.MiR-206 participates in epileptic brain injury by modulating neuronal survival and synaptic plasticity downstream of the BDNF/TrkB cascade.
- Targeting miR-206 can simultaneously ameliorate seizure activity, neuronal degeneration, and cognitive dysfunction in experimental TLE. MiR-206 may serve as a potential epigenetic therapeutic target for treating refractory temporal lobe epilepsy.

**Abstract:**

**Objectives**: Temporal lobe epilepsy (TLE) causes spontaneous seizures, hippocampal neuronal loss, and cognitive impairment. This study investigated whether miR-206 inhibition attenuates seizures and cognitive dysfunction by activating the BDNF/TrkB cascade in mice with pilocarpine-induced TLE. **Methods**: Fifty TLE-model C57BL/6N mice were divided into five groups. MiR-206 antagomir or a negative control was stereotaxically injected into the hippocampus. ANA-12, a TrkB inhibitor, was given intraperitoneally. We detected miR-206 with RT-qPCR. EEG was used to assess seizure activity. Spatial cognition was measured via the Morris water maze. Neuronal injury was evaluated using HE and Nissl staining. Target protein levels were quantified by Western blotting. Hippocampal miR-206 was elevated in TLE mice. **Results**: MiR-206 antagomir lowered miR-206 expression. It prolonged seizure latency. It reduced seizure frequency and duration. It restored spatial learning and memory function. It relieved CA3 neuronal loss and up-regulated BDNF/TrkB, SYP, and PSD95. ANA-12 abolished these protective phenotypes. **Conclusions**: MiR-206 inhibition confers anti-seizure, neuroprotective, and cognition-improving effects. These benefits depend on functional BDNF/TrkB signalling. miR-206 is a potential therapeutic target for TLE.

## 1. Introduction

Epilepsy is a common and severe neurological disorder characterized by recurrent spontaneous seizures [1]. It affects millions of individuals worldwide [2]. Despite advances in clinical treatment, the precise molecular mechanisms underlying epileptogenesis remain poorly understood. This situation hinders the development of effective therapeutic strategies [3]. Epilepsy patients bear a heavy disease burden with poor prognosis and increased risk of complications [4]. It is urgent to identify novel therapeutic targets and molecular pathways.

MicroRNAs (miRNAs) are small noncoding RNAs of approximately 22 nucleotides in length [5]. They are widely involved in the onset and progression of various human diseases, including neurological disorders [6]. MiRNAs exert critical regulatory functions by modulating gene expression at the post-transcriptional level. They mainly bind to the 3′-untranslated region (3′UTR) of target mRNAs to suppress translation or promote mRNA degradation [7]. Mounting studies have confirmed the vital roles of miRNAs in neurological homeostasis and pathological processes. These studies make miRNAs promising candidates for disease diagnosis and treatment [8,9,10,11,12,13,14,15].

BDNF is a neurotrophin predominantly expressed in the central nervous system (CNS) [16]. It plays an indispensable role in normal CNS development, neuronal growth and survival, neural repair, and synaptic plasticity [17]. These functions are closely associated with epileptogenesis and epilepsy progression [18]. The regulatory roles of miRNAs in epilepsy have attracted increasing attention in recent years. Multiple miRNAs have been identified as critical modulators of epileptogenesis [19,20]. Bioinformatic analysis using the TargetScan online database predicted a potential binding site between miR-206 and BDNF. MiR-206 has been proposed as a pivotal molecule involved in epilepsy pathogenesis [21]. The miR-206/BDNF regulatory axis has been implicated in several central nervous system disorders [22,23,24,25], yet whether miR-206 modulates the BDNF/TrkB pathway in temporal lobe epilepsy and whether this modulation is functionally relevant remains unclear. This study addresses this gap using pharmacological TrkB blockade for rescue validation. However, the specific regulatory mechanism of miR-206 in pilocarpine-induced temporal lobe epilepsy (TLE) via the BDNF/TrkB signaling pathway remains largely uncharacterized. This study is the first to demonstrate that miR-206 antagomir alleviates seizures, improves cognitive function, and protects hippocampal neurons by activating the BDNF/TrkB pathway in a pilocarpine-induced chronic epilepsy mouse model, filling the gap in the mechanistic link between miR-206 and epilepsy-related synaptic plasticity and neuronal injury. It is reasonable and necessary to investigate the molecular mechanism of miR-206 in epilepsy and evaluate its potential as a novel anti-epileptic therapeutic target.

Tyrosine kinase receptor B (TrkB) is a specific receptor for BDNF. BDNF can activate the TrkB receptor to initiate multiple downstream signaling pathways [26]. The BDNF/TrkB signaling pathway plays a crucial role in regulating neuronal proliferation and differentiation, apoptosis, injury repair, neurotransmitter release, and synaptic plasticity [27]. Synapses are the basic units for information transmission between neurons. Synaptic plasticity is an important physiological basis for learning and memory [28]. Synaptophysin (SYP) and postsynaptic density protein 95 (PSD95) are key proteins that regulate synaptic plasticity [29,30]. SYP serves as a marker for the presynaptic membrane, and PSD95 acts as a marker for the postsynaptic membrane. Abnormal expression of either protein can lead to impaired synaptic plasticity [31,32,33].

Based on the above evidence, we hypothesized that miR-206 participates in epilepsy by regulating BDNF expression. We used a mouse model of pilocarpine-triggered temporal lobe epilepsy (TLE) in the current work. This model is a classic model that mimics clinical epilepsy [16,17,18,19]. This work sought to investigate the biological roles of miR-206 in epilepsy. We also aimed to uncover its underlying molecular mechanism. Our results elucidate the protective effects conferred by miR-206 antagomir in epilepsy. They also uncover the corresponding molecular signaling pathway. These observations offer theoretical support for developing innovative targeted therapies against epilepsy.

## 2. Materials and Methods

### 2.1. Chemicals

Pilocarpine, scopolamine, and diazepam were obtained from Sigma (St. Louis, MO, USA). MiR-206 antagomir and miR-206 antagomir control were obtained from Hippobio (Huzhou, China). ANA-12 was obtained from Beyotime (Shanghai, China). Antibodies against GAPDH, BDNF, TrkB, SYP, and PSD95 were obtained from Proteintech (Wuhan, China). RT-qPCR kits were obtained from Vazyme (Nanjing, China). HE staining kits and Nissl staining kits were obtained from Servicebio (Wuhan, China).

### 2.2. Subjects

Fifty 8-week-old C57BL/6N male mice weighing 20–24 g were provided by Beijing Vital River Laboratory Animal Technology. All protocols were approved by the Institutional Animal Ethics Committee of Dalian Medical University. All procedures were conducted in accordance with the Guide for the Care and Use of Laboratory Animals. All mice were housed in a specific pathogen-free (SPF) animal room. The ambient temperature was controlled at 22 ± 2 °C, and the relative humidity was 50–60%. A 12 h/12 h light–dark cycle was implemented with lights on at 8:00 and lights off at 20:00. All animals were provided with standard laboratory chow and drinking water ad libitum. Before the formal experiment, all mice were housed five per cage for 7 days of acclimatization. After experimental modeling, mice were reared individually in single cages until the end of the experiment.

### 2.3. Experimental Timeline

Day 0: Establishment of the pilocarpine-induced chronic epilepsy mouse model

Days 1–7: Daily intraperitoneal injection of ANA-12

Day 1: Hippocampal stereotaxic injection of miR-206 antagomir (ANA-12 intraperitoneal injection 1 h before the procedure)

Day 8: EEG electrode implantation

Days 13–23: Continuous EEG monitoring (1 h/day per mouse)

Days 24–28: Morris Water Maze test

Day 28: Brain tissue collection

### 2.4. Pilocarpine-Induced Chronic Epilepsy Model

To establish the pilocarpine-induced chronic epilepsy model, all mice received an intraperitoneal (i.p.) injection of scopolamine at 1 mg/kg body weight for pretreatment. This pretreatment blocked peripheral cholinergic responses and eliminated the peripheral side effects of pilocarpine. After 30 min, epileptic seizures were induced by i.p. administration of pilocarpine at a dose of 325 mg/kg. After pilocarpine injection, mice were continuously monitored to observe the onset and progression of seizure behaviors. Seizure severity was evaluated and graded according to the modified Racine scale. Grade 1 was facial twitching. Grade 2 was head nodding. Grade 3 was unilateral forelimb clonus. Grade 4 was bilateral forelimb clonus with rearing. Grade 5 was generalized tonic–clonic seizures with falling. Only mice that developed sustained grade 4–5 seizures were included in subsequent experiments. To terminate persistent epileptic seizures and reduce excessive neuronal excitotoxicity and animal mortality, diazepam at 10 mg/kg was administered via i.p. injection 60 min after the onset of sustained stage 4–5 seizures. Diazepam at 10 mg/kg reduces the mortality rate of experimental mice. It also ensures the successful establishment of a stable chronic epilepsy model. Mice that failed to exhibit typical epileptic seizures or died during modeling were excluded from the study.

### 2.5. Animal Grouping

TLE mice were randomly divided into five groups with 10 mice per group.

Group 1/TLE group: PILO-induced epileptic mice.

Group 2/TLE + miR-206 AC group: PILO-induced epileptic mice injected with miR-206 antagomir negative control.

Group 3/TLE + miR-206 A group: PILO-induced epileptic mice injected with miR-206 antagomir.

Group 4/TLE + ANA-12 group: PILO-induced epileptic mice injected with ANA-12.

Group 5/TLE + miR-206 A + ANA-12 group: PILO-induced epileptic mice injected with both miR-206 antagomir and ANA-12.

### 2.6. Surgeries and Treatment

Mice in groups 2, 3, and 5 were anesthetized with a gas anesthesia machine. Mice were anesthetized with isoflurane by inhalation at 5% for the induction phase and 2.5% for maintenance. They were placed in a stereotaxic frame. After scalp incision and exposure of the skull, bilateral hippocampal injection sites were located and marked. The coordinates were AP: −2.0 mm, ML: ±1.5 mm, DV: −1.8 mm from bregma. Burr holes were drilled using a dental drill. MiR-206 antagomir was used to knock down miR-206 expression. MiR-206 antagomir at 1 nmol per side was microinjected into the hippocampal region of mice in groups 3 and 5. MiR-206 antagomir control at 1 nmol per side was microinjected into the hippocampal region of mice in group 2. All microinjections were performed at a constant injection rate of 0.2 ul/min. The total injection volume was 1.0 ul at 1 nmol/ul per hemisphere. The needle was retained for 5 min after injection to prevent reflux and then withdrawn. The scalp was sutured. Mice in groups 4 and 5 received intraperitoneal injections of ANA-12 for 7 consecutive days. ANA-12 is a TrkB receptor antagonist used at 0.5 mg/kg. Injections were given 1 h before the start of the miR-206 antagomir injection.

### 2.7. SRS Latency

In this study, the latency of spontaneous recurrent seizures in the pilocarpine-induced chronic epileptic mouse model was evaluated by continuous behavioral observation. This evaluation followed the standard paradigm for epileptic animal models. The day on which status epilepticus was successfully terminated was defined as post-SE day 0. All experimental mice were housed individually in standard laboratory cages after SE induction. This housing condition avoided social stress and seizure suppression caused by group housing.

Formal behavioral monitoring started on post-SE day 0 and lasted until post-SE day 28. To minimize observational omission and ensure stable detection conditions, fixed-time observation windows were adopted daily. The windows were 9:00–10:00 and 14:00–15:00. Each mouse received 60 min of undisturbed in-cage behavioral observation per time window. No grabbing, cage moving, or other external interference that may cause stress was allowed. To further reduce the omission of night seizures, rapid cage inspections of 5–10 min each were performed three times every night. These inspections observed the presence of generalized tonic–clonic seizures.

SRS judgment was strictly based on the modified Racine scale. A valid spontaneous seizure was defined as a behavioral seizure of Racine grade III or higher that lasted for no less than 10 consecutive seconds. Specifically, Racine grade III was characterized by forelimb clonus and unstable standing posture. Grade IV was rearing accompanied by persistent forelimb clonus. Grade V was rearing followed by falling and generalized tonic–clonic convulsions. Normal spontaneous behaviors, including grooming, temporary shaking, exploratory walking, and jumping, were excluded to avoid false-positive judgment.

The SRS latency of each mouse was recorded as the interval in days from post-SE D0 to the day when the first valid SRS was observed. Mice that did not exhibit Racine grade III or higher spontaneous seizures within 28 days after SE were regarded as non-epileptic mice. Behavioral monitoring is a classic and widely recognized method for evaluating SRS latency in pilocarpine epileptic models. Limited by manual observation conditions, there may be a slight risk of missed detection of individual seizures. This situation is a common methodological limitation of behavioral phenotyping in epilepsy animal experiments.

### 2.8. EEG Installation and Monitoring

A commercially available three-channel wired recording system was used for electrophysiological signal acquisition. The system included 2 EEG channels and 1 EMG channel (Pinnacle Technology Inc., Lawrence, KS, USA). Four stainless steel screw electrodes were anchored on the mouse skull for dual-channel EEG signal recording. EEG signals were band-pass filtered with a high-pass cutoff at 1.0 Hz and a low-pass cutoff at 15 kHz. EMG signals were band-pass filtered with a fixed high-pass cutoff of 10 Hz and a low-pass cutoff of 15 kHz. The sampling rate was set within the range of 200 Hz to 2000 Hz for the three-channel recording configuration.

EEG electrodes were implanted in all groups. Mice were anesthetized with an isoflurane gas machine, and their heads were fixed on a brain stereotaxic instrument. The hair on the backs of the mice’s heads was removed. The surgical area was disinfected, and the skin was cut. A small amount of H_2_O_2_ was applied to the skull to make the cranial sutures clear. Specifically, the two anterior screws for EEG1 recording were positioned at AP +2.0 mm and ML ±1.5 mm relative to the bregma. The two posterior screws for EEG2 recording were placed at AP −4.0 mm and ML ±1.5 mm. Two flexible EMG recording electrodes were bilaterally inserted into the cervical musculature of mice to capture myoelectric activity. The electrode assembly and cranial headmount were firmly secured and sealed on the skull surface with dental cement.

To avoid circadian rhythm interference, each mouse received 1 h of fixed-time daily EEG monitoring in a quiet, temperature-controlled, independent recording chamber. External strong light, loud noise, and mechanical disturbance were strictly prohibited. These measures reduced behavioral artifacts and invalid signals. All EEG and EMG signals were synchronously acquired and recorded using the software Pinnacle Sirenia Acquisition (version 2.1.0, Pinnacle Technology, Lawrence, KS, USA). Epileptic burst discharges were defined as discrete high-amplitude clustered spike-wave or polyspike-wave complexes that lasted for 3 s or longer. These complexes were clearly distinguishable from stable baseline EEG activity. Only qualified burst discharge events with significant waveform aggregation and abnormal electrical excitation were included for statistical analysis. The core quantitative indicators focused on epileptic burst discharges in this study were as follows. (1) SRS frequency: total number of burst discharges. This was the total count of valid epileptic burst discharge events within the 1-h daily monitoring period. (2) SRS duration: average burst discharge duration. This was the mean sustained time of each epileptic burst discharge episode. All analyses of epileptic burst discharge data were independently performed by two researchers in a blinded manner. This procedure eliminated subjective evaluation bias. Artifactual signals induced by mouse struggling, grooming, equipment vibration, and environmental electromagnetic interference were manually identified and excluded from statistical analysis. All valid burst discharge parameters were summarized and averaged. This process ensured the accuracy, stability, and repeatability of electrophysiological results.

### 2.9. Morris Water Maze Test

The Morris Water Maze test was performed to evaluate the spatial learning and memory ability of mice. The Morris Water Maze consists of a cylindrical water tank with a diameter of 100 cm and a height of 50 cm, a platform, cameras, and video analysis software. By setting software parameters, the water tank image was evenly divided into 4 quadrants. In each quadrant, a conspicuous circle, triangle, square, or star was placed on the water surface above the tank wall as a reference. These markers remained unchanged throughout the experiment. A random quadrant was selected. A transparent circular escape platform with a diameter of 10 cm and a height of 25 cm was placed in the center of this quadrant. Warm water at 22 °C was injected into the water tank. The water level was 2 cm higher than the platform. Flour was added to the tank and mixed evenly to make the platform completely hidden. The surrounding area of the water tank was covered with curtains to avoid interference from sound and light to the mice. The camera on the top of the tank recorded the movement trajectory of the mice.

The place navigation experiment was used to study the spatial learning ability of mice. Mice with strong memory had a shorter escape latency. Mice were trained 4 times a day for a fixed period of 4 days. A camera was placed above the water tank. The software was used to record the movement trajectory of the mice. The escape latency of the mice in finding the platform was recorded. Mice faced the tank wall and turned their backs to the center of the water tank. They were placed in the water in each quadrant. The mice searched for the platform hidden under the water surface. If the mice found the platform within 90 s, they stayed on the platform for 15 s. The time taken to find and climb onto the platform was recorded as the escape latency. If the mice did not find the escape platform within 90 s, the operator guided them to the platform. They stayed on the platform for 15 s, and the latency was recorded as 90 s. The average latency of the mice during the 4 training sessions each day was used to evaluate the learning performance of the mice on that day.

The spatial exploration experiment was used to detect the spatial memory ability of mice. Mice with strong memory swam for a longer time in the original platform quadrant. They also swam more times in the area of the original platform. Mice with impaired memory swam aimlessly and swam fewer times in the area of the original platform. Twenty-four hours after the end of the place navigation experiment, the hidden escape platform was removed. The quadrant opposite the platform was selected as the entry point for the mice into the water. Mice were placed facing the tank wall and were not interfered with during swimming. The number of times the mice crossed the platform area within 90 s and the swimming time in the platform quadrant were recorded.

### 2.10. Mouse Brain Tissue Samples

After the completion of the Morris Water Maze test, all mice were anesthetized with 2% isoflurane. Adequate anesthetic depth was verified by the loss of corneal and pedal reflexes. Under complete anesthesia, the mice immediately received transcardial perfusion with ice-cold 0.9% sterile normal saline. Perfusion was performed until the liver turned pale and the outflow became clear. This process removed intravascular blood components. The brain tissues were rapidly isolated. Half of the brain tissues were collected for subsequent molecular biology experiments. The hippocampal region was separated for Western blot and qPCR analyses. The other half was reserved for histological observation. For histological analysis, mouse brain tissues were fixed in 4% paraformaldehyde, dehydrated, and embedded in paraffin. Serial 5 μm-thick coronal sections were prepared for subsequent histological staining.

### 2.11. HE Staining and Nissl Staining

For HE staining, brain tissue sections were dewaxed using xylene. They were stained with hematoxylin and eosin, dehydrated with absolute ethanol, and sealed with neutral gum.

For Nissl staining, dewaxed and dehydrated brain tissue sections were stained with Cresyl violet acetate. They were then washed with distilled water. These sections were dehydrated with ethanol and xylene and sealed with neutral gum.

HE and Nissl staining were performed to observe neuronal morphology in the hippocampal CA3 region with 5 mice per group. For each mouse, 3 coronal sections were randomly selected. Visual fields in the CA3 region of each section were captured using an Olympus BX53 upright optical microscope (Olympus Corporation, Tokyo, Japan) at 200× and 400× magnifications.

Nissl staining was used to count the number of neurons in the hippocampal CA3 region in 5 mice per group. ImageJ software (version 1.53t, National Institutes of Health, Bethesda, MD, USA; RRID:SCR_003070) was used for neuronal counting and quantitative analysis. Intact surviving neurons were defined as cells with regular arrangement, clear nuclear structure, uniform staining, and abundant cytoplasmic Nissl granules. In contrast, shrunken, atrophic, pyknotic, or severely deformed neurons with blurred structure and lost Nissl granules were excluded from statistics. The average number of surviving neurons per mouse was calculated. This value evaluated the degree of hippocampal neuronal loss and structural damage.

### 2.12. RT-qPCR

RT-qPCR was used to detect the level of miR-206 in the hippocampus. Total RNA was extracted using the Total RNA Isolation Kit. RNA was reverse-transcribed into cDNA using cDNA Synthesis kits. RT-qPCR was carried out using SYBR qPCR Mix kits on a QuantStudio 5 Dx Real-Time PCR system. U6 served as the internal reference for miR-206. Measurement data were quantified using the 2^−(△△Ct)^ method.

### 2.13. Western Blotting

Western blotting was performed to detect the expression levels of BDNF, TrkB, SYP, and PSD95 proteins in mouse hippocampal tissues. Total protein was extracted using ice-cold RIPA lysis buffer (Epizyme Biotech, Shanghai, China) supplemented with protease inhibitors (Epizyme Biotech, Shanghai, China). The protein concentration of each sample was determined via the BCA protein quantification method. For electrophoresis, a total of 30 μg protein with a 10 μL loading volume per sample was separated by 10% sodium dodecyl sulfate-polyacrylamide gel electrophoresis (SDS-PAGE). The separated proteins were then electrophoretically transferred onto polyvinylidene fluoride (PVDF) membranes. For non-specific binding blocking, the membranes were incubated with 5% (*w*/*v*) skim milk powder dissolved in Tris-buffered saline containing 0.1% Tween-20 (TBST) at room temperature (25 °C) for 2 h with gentle shaking.

All primary and secondary antibodies used in this study were purchased from Proteintech Group (Wuhan, China). The membranes were incubated with diluted primary antibodies against BDNF (Cat. No. 28205-1-AP, 1:1000 dilution), TrkB (Cat. No. 13129-1-AP, 1:3000 dilution), SYP (Cat. No. 17785-1-AP, 1:3000 dilution), PSD95 (Cat. No. 20665-1-AP, 1:2000 dilution) and GAPDH (Cat. No. 81640-5-RR, 1:5000 dilution) overnight at 4 °C. After thorough washing with TBST, the membranes were incubated with HRP-conjugated secondary antibody (Cat. No. RGAR001, 1:5000 dilution) at 25 °C for 1 h. Protein bands were visualized using an enhanced chemiluminescence (ECL) ultra-sensitive luminescence reagent (Shanghai Epizyme Biotech) and imaged with a Western blot imaging system. The grayscale intensity of each protein band was quantified using ImageJ software. The relative expression level of each target protein was normalized to the internal reference GAPDH for statistical analysis. All experiments were repeated at least three times independently.

### 2.14. Statistical Analysis

Measurement data were presented as mean ± SEM. n represents independent biological replicates derived from individual mice. Each biological replicate was assessed with 3 internal technical replicates to reduce experimental variation. Technical replicates were not treated as independent statistical units. All measurement data were subjected to normality and homogeneity of variance tests. Comparisons between groups of measurement data were conducted using one-way ANOVA. Comparisons of water maze escape latency used repeated measures ANOVA. When homogeneity of variance was achieved, pairwise comparisons were performed using the LSD test. When heterogeneity of variance was observed, Tamhane’s T2 test was used. All statistical analyses and graph manipulation were conducted using GraphPad Prism 8. A *p*-value < 0.05 indicates a statistically significant difference. (ns *p* > 0.05. * *p* < 0.05. ** *p* < 0.01. *** *p* < 0.001).

## 3. Results

### 3.1. Hippocampal miR-206 Level

**Figure 1:**
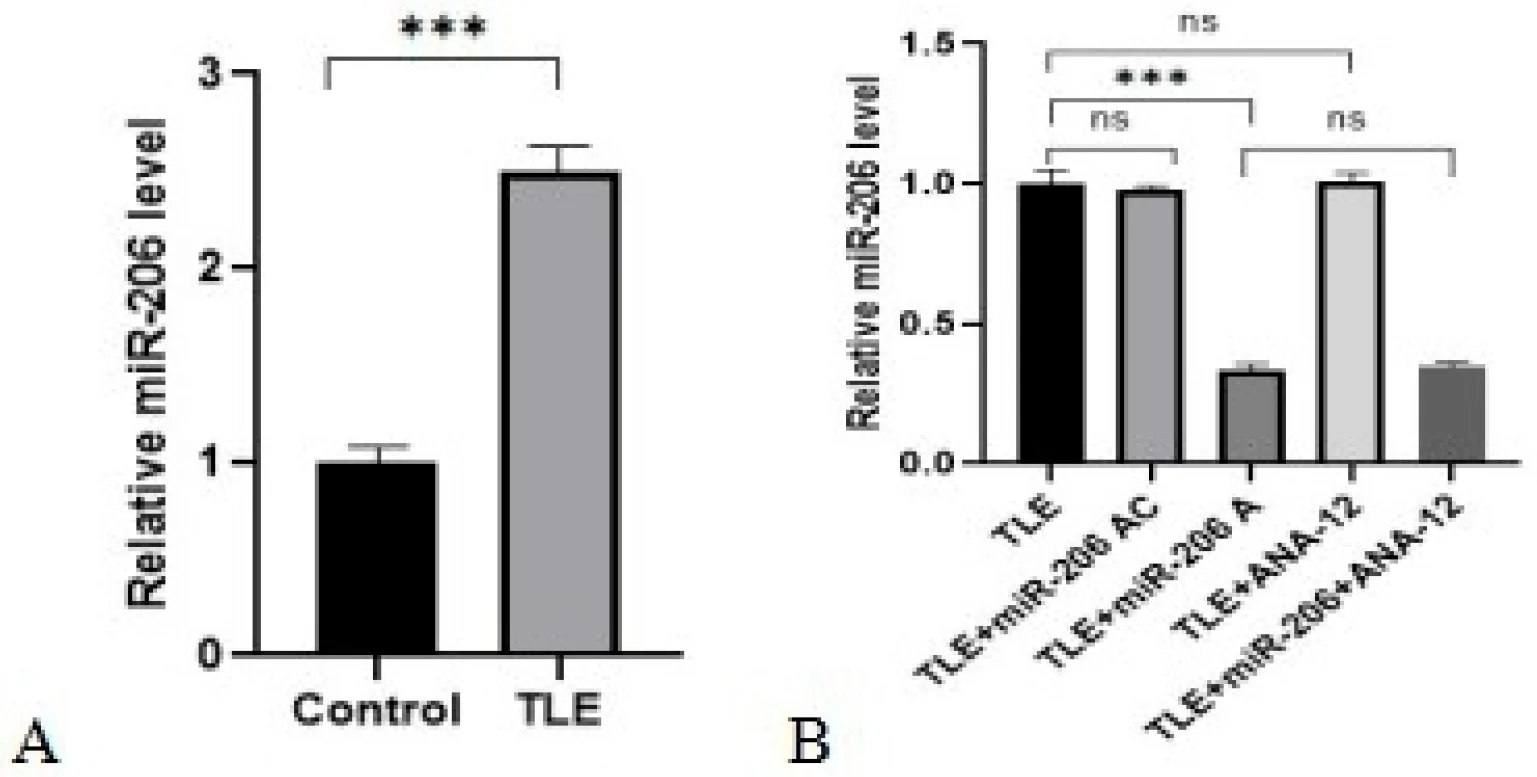
Expression of miR-206 in the mouse hippocampus detected by RT-qPCR. (**A**) Relative miR-206 expression in the control group and TLE model group. (**B**) Relative miR-206 expression in five TLE mouse groups. The relative expression of miRNA was normalized to U6 snRNA. Data are presented as mean ± SEM *n* = 5 independent biological replicates per group, each replicate derived from one individual mouse with 3 technical replicates for each sample. Statistical significance was determined by one-way ANOVA followed by the LSD test for homogeneous variances or Tamhane’s T2 test for heterogeneous variances. (ns *p* > 0.05, *** *p* < 0.001).

As shown in Figure 1A and Table A1, miR-206 was markedly upregulated in the TLE group compared with normal control mice. The baseline data of normal control mice were derived from our previous study (*n* = 10). Normal-control mice underwent the same RNA isolation and RT-qPCR procedures as TLE model animals. The baseline data of normal control mice (*n* = 10) are provided in Supplementary Figure S1 and Supplementary Table S1 of the present manuscript.

As illustrated in Figure 1B and Table A2, miR-206 expression was significantly downregulated in the TLE + miR-206 A group compared with the TLE group. In contrast, no obvious difference in miR-206 level was observed in the TLE + miR-206 AC group and the TLE + ANA-12 group compared with the TLE group. In addition, there was no significant difference in miR-206 expression between the TLE + miR-206 A group and the TLE + miR-206 A + ANA-12 group.

### 3.2. EEG Results

#### 3.2.1. Spontaneous Recurrent Seizures (SRS) Latency

**Figure 2:**
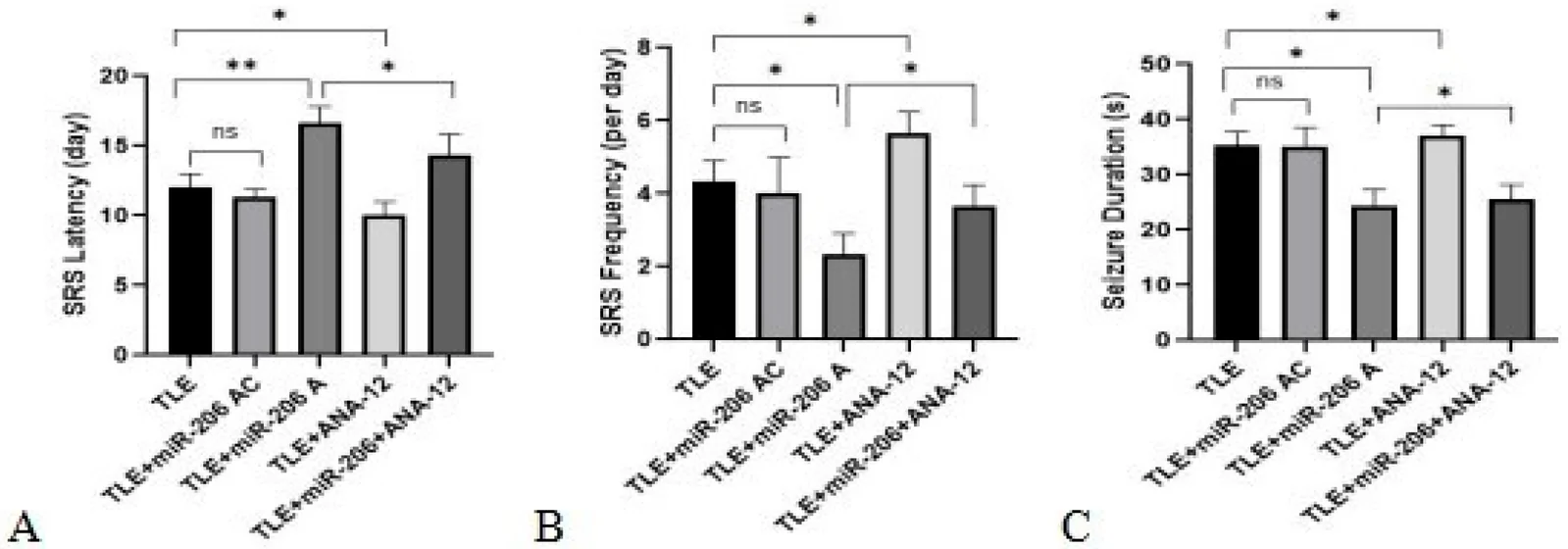
Effects of miR-206 antagomir and ANA-12 on SRS in epileptic mice evaluated by EEG. (**A**) Latency to the first spontaneous recurrent seizure. (**B**) Frequency of epileptic burst discharges within 1 h. (**C**) Average duration of epileptic burst discharges. Epileptic burst discharges were defined as high-amplitude clustered spike-wave or polyspike-wave complexes lasting ≥3 s. Data are presented as mean ± SEM (*n* = 10 per group). Statistical significance was determined by one-way ANOVA followed by the LSD test for homogeneous variances or Tamhane’s T2 test for heterogeneous variances. (ns *p* > 0.05, * *p* < 0.05, ** *p* < 0.01).

As shown in Figure 2A and Table A3, SRS latency in the TLE + miR-206 AC group showed no significant difference compared with the TLE group. SRS latency was significantly prolonged in the TLE + miR-206 A group compared with the TLE group. SRS latency was significantly shortened in the TLE + ANA-12 group compared with the TLE group. SRS latency was significantly shortened in the TLE + miR-206 A + ANA-12 group compared with the TLE + miR-206 A group.

#### 3.2.2. SRS Frequency

As shown in Figure 2B and Table A3, SRS frequency in the TLE + miR-206 AC group showed no significant difference compared with the TLE group. SRS frequency was significantly decreased in the TLE + miR-206 A group compared with the TLE group. SRS frequency was significantly increased in the TLE + ANA-12 group compared with the TLE group. SRS frequency was significantly increased in the TLE + miR-206 A + ANA-12 group compared with the TLE + miR-206 A group.

#### 3.2.3. SRS Duration

As shown in Figure 2C and Table A3, SRS duration in the TLE + miR-206 AC group showed no significant difference compared with the TLE group. SRS duration was significantly shortened in the TLE + miR-206 A group compared with the TLE group. SRS duration was significantly prolonged in the TLE + ANA-12 group compared with the TLE group. SRS duration was significantly prolonged in the TLE + miR-206 A + ANA-12 group compared with the TLE + miR-206 A group.

### 3.3. Morris Water Maze Test Results

#### 3.3.1. Place Navigation Test

**Figure 3:**
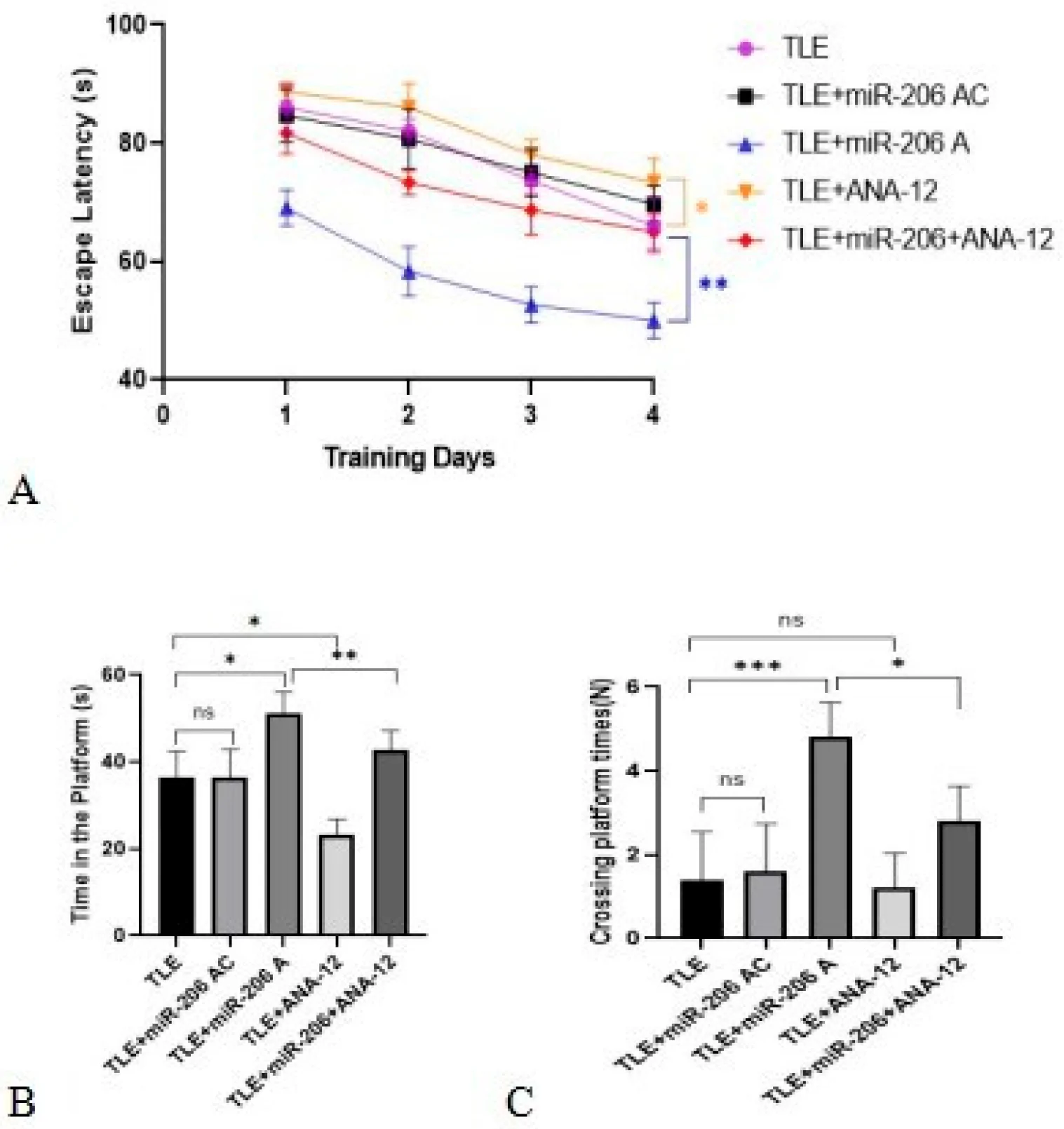
Mice in the Morris Water Maze. (**A**) Escape latency of mice in the place navigation test. Mice were trained for 4 consecutive days. Significant time effect, group effect, and group–time interaction were detected by repeated-measures two-way ANOVA (all *p* < 0.05). Post hoc multiple comparisons were performed for intergroup differences at each training day. (**B**) Time spent in the platform quadrant in the spatial probe test. (**C**) Number of platform crossings in the spatial probe test. Statistical significance was determined by one-way ANOVA followed by the LSD test for homogeneous variances or Tamhane’s T2 test for heterogeneous variances. Data are presented as mean ± SEM (*n* = 10 per group). (ns *p* > 0.05, * *p* < 0.05, ** *p* < 0.01, *** *p* < 0.001).

As shown in Figure 3A and Table A4, the escape latency of all groups gradually decreased after 4 days of training. The escape latency in the TLE + miR-206 AC group showed no significant difference compared with the TLE group. The escape latency in the TLE + miR-206 A group was significantly shortened compared with the TLE group. The escape latency in the TLE + miR-206 A + ANA-12 group was significantly prolonged compared with the TLE + miR-206 A group.

#### 3.3.2. Spatial Probe Test

As shown in Figure 3B and Table A5, there was no significant difference in time spent in the platform quadrant between the TLE group and the TLE + miR-206 AC group. The time spent in the platform quadrant was markedly prolonged in the TLE + miR-206 A group compared with the TLE group. The time spent in the platform quadrant was significantly shortened in the TLE + miR-206 A + ANA-12 group compared with the TLE + miR-206 A group.

As shown in Figure 3C and Table A5, there was no significant difference in the number of platform crossings between the TLE group and the TLE + miR-206 AC group. The number of platform crossings was significantly increased in the TLE + miR-206 A group compared with the TLE group. The number of platform crossings was significantly reduced in the TLE + miR-206 A + ANA-12 group compared with the TLE + miR-206 A group.

### 3.4. HE Staining Results

**Figure 4:**
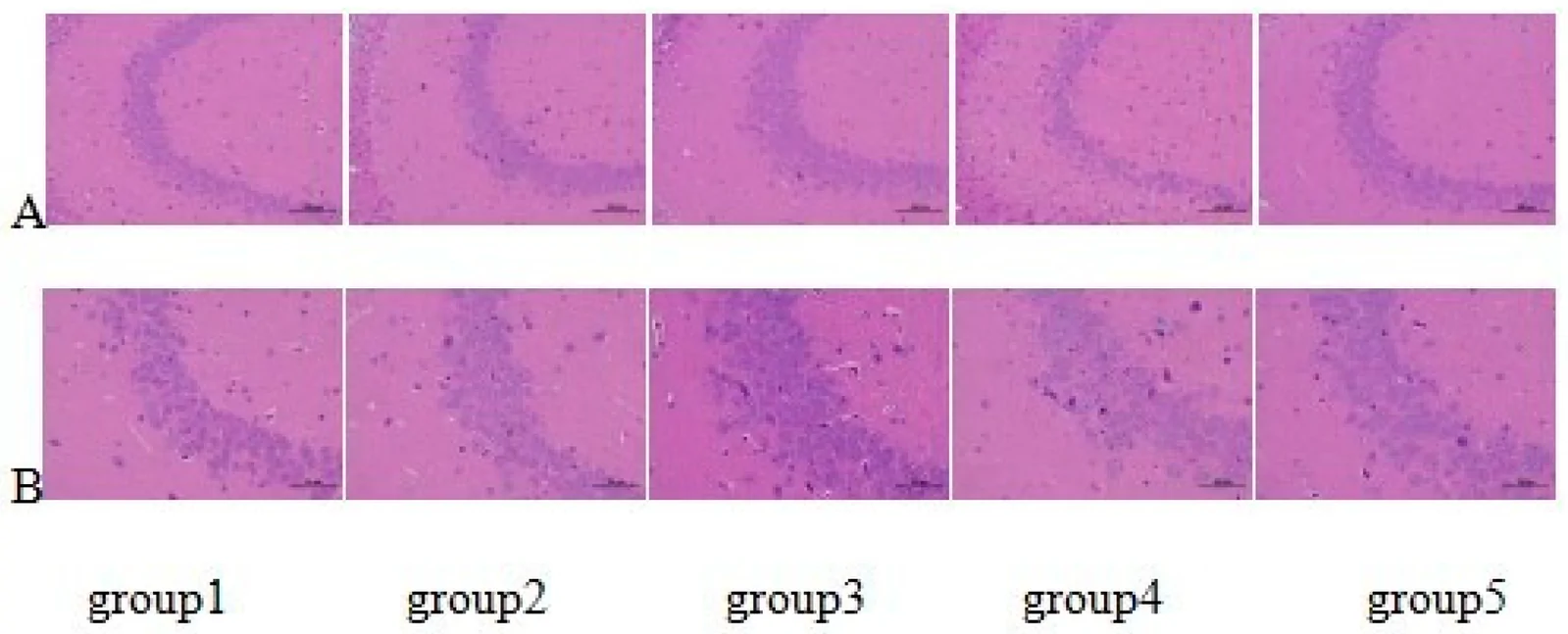
HE staining of the hippocampal CA3 region in pilocarpine-induced epileptic mice. (**A**) Representative images at 200× magnification; scale bar = 100 um. (**B**) Representative images at 400× magnification; scale bar = 50 um. Neuronal morphology and arrangement were observed in five groups. *n* = 5 independent biological replicates per group each replicate derived from one individual mouse with 3 technical counting fields for each animal.

As shown in Figure 4, all five groups exhibited a disorganized arrangement of pyramidal cells and obvious neuronal loss in the hippocampal CA3 region. The TLE + miR-206 A group showed markedly improved cellular arrangement and reduced neuronal pyknosis and cytoplasmic vacuolation compared with the TLE model group. These results indicate alleviation of neuronal damage. The TLE + ANA-12 group displayed more severe pathological changes compared with the TLE + miR-206 A + ANA-12 group. These changes included more disordered cell arrangement, widespread nuclear pyknosis, and aggravated vacuolar degeneration. HE staining results morphologically confirm that miR-206 alleviates neuronal injury in the hippocampal CA3 region of temporal lobe epilepsy. ANA-12 blocks this neuroprotective effect.

### 3.5. Nissl Staining Results

**Figure 5:**
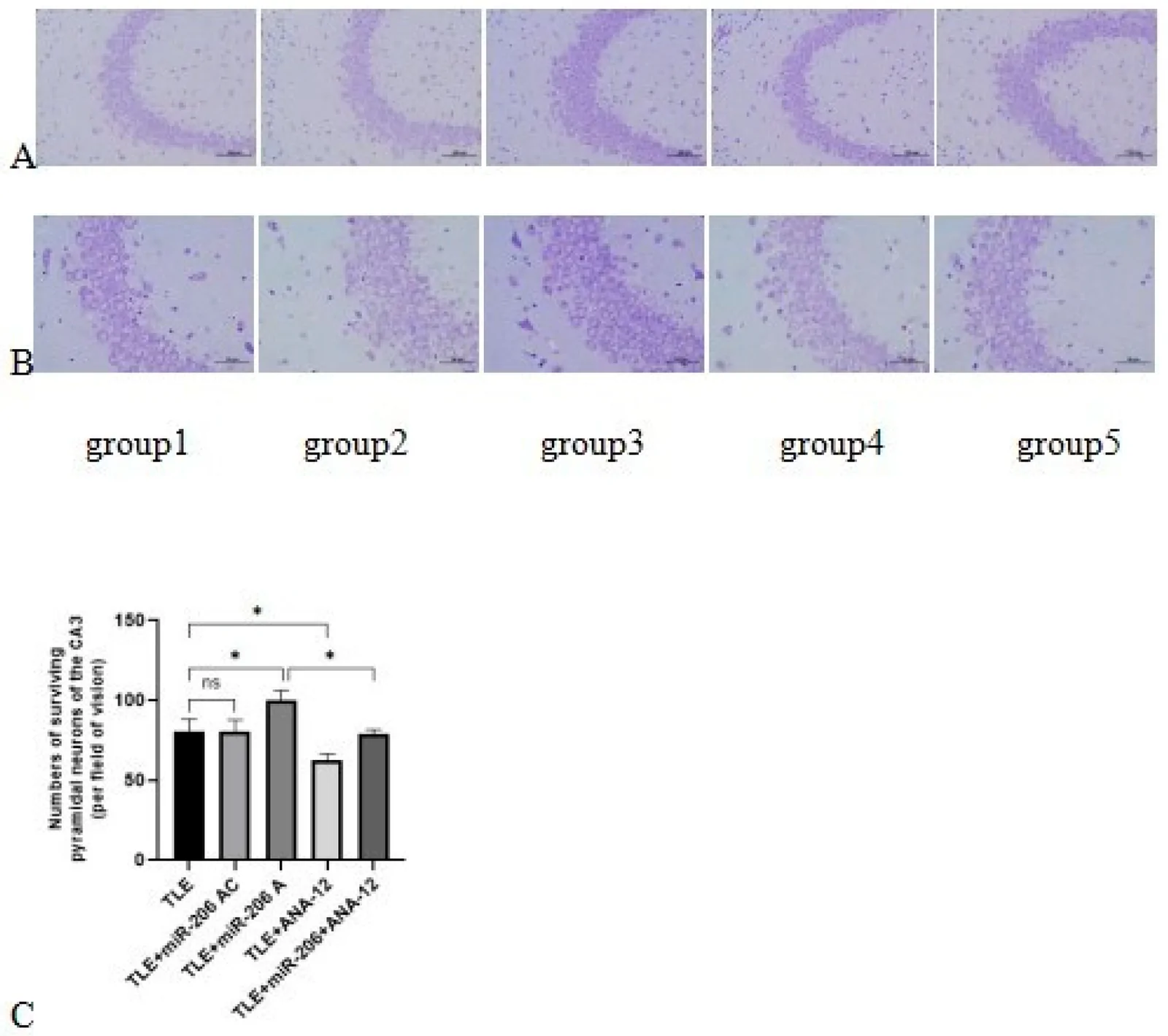
Nissl staining and quantitative analysis of hippocampal CA3 neurons in epileptic mice. (**A**) Representative images at 200× magnification; scale bar = 100 μm. (**B**) Representative images at 400× magnification; scale bar = 50 μm. (**C**) Quantitative analysis of surviving neuron density in the CA3 region. Data are presented as mean ± SEM *n* = 5 independent biological replicates per group each replicate derived from one individual mouse with 3 technical counting fields for each animal. Statistical significance was determined by one-way ANOVA followed by the LSD test for homogeneous variances or Tamhane’s T2 test for heterogeneous variances. (ns *p* > 0.05, * *p* < 0.05).

As shown in Figure 5A,B, all five groups exhibited partial neuronal loss, sparse arrangement, nuclear pyknosis, unclear nucleoli, and reduced Nissl bodies in the hippocampal CA3 region. As shown in Table A6, the number of neurons in the TLE + miR-206 AC group showed no significant difference compared with the TLE group. The number of neurons in the TLE + miR-206 A group was significantly increased compared with the TLE group. The number of neurons in the TLE + ANA-12 group was significantly decreased compared with the TLE group. The number of neurons in the TLE + miR-206 A + ANA-12 group was significantly decreased compared with the TLE + miR-206 A group. Nissl staining results morphologically confirm that miR-206 alleviates neuronal injury in the hippocampal CA3 region of temporal lobe epilepsy. ANA-12 blocks this neuroprotective effect.

### 3.6. Western Blotting Results

**Figure 6:**
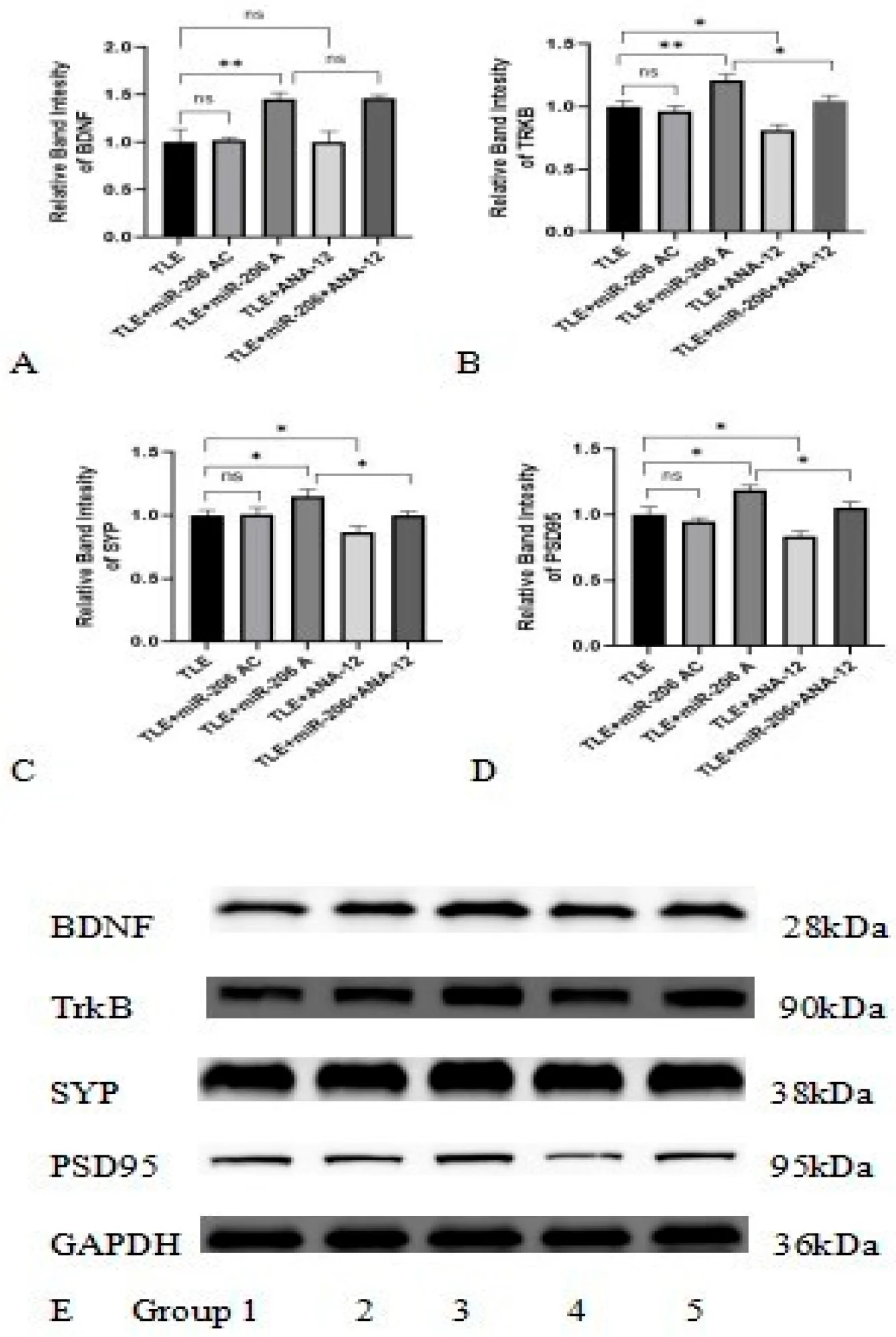
Protein expression levels of BDNF, TrkB, SYP, and PSD95 in the hippocampus of epileptic mice. (**A**–**D**) Quantitative analysis of relative protein expression levels normalized to GAPDH. (**E**) Representative Western blot bands of target proteins. Data are presented as mean ± SEM *n* = 5 independent biological replicates per group each replicate derived from one individual mouse with three technical replicate west-ern blot runs for each sample. Statistical significance was determined by one-way ANOVA followed by the LSD test for homogeneous variances or Tamhane’s T2 test for heterogeneous variances. (ns *p* > 0.05, * *p* < 0.05, ** *p* < 0.01).

As shown in Figure 6A,E and Table A7, the BDNF in the TLE + miR-206 A group was significantly increased compared with the TLE group. The BDNF in the TLE + miR-206 AC group showed no significant difference compared with the TLE group. The BDNF in the TLE + ANA-12 group showed no significant difference compared with the TLE group. The BDNF in the TLE + miR-206 A + ANA-12 group showed no significant difference compared with the TLE + miR-206 A group.

As shown in Figure 6B–E and Table A7, TrkB, SYP, and PSD95 in the TLE + miR-206 A group were significantly increased compared with the TLE group. The TrkB, SYP, and PSD95 in the TLE + miR-206 AC group showed no significant difference compared with the TLE group. TrkB, SYP, and PSD95 in the TLE + ANA-12 group were significantly decreased compared with the TLE group. TrkB, SYP, and PSD95 in the TLE + miR-206 A + ANA-12 group were significantly decreased compared with the TLE + miR-206 A group.

## 4. Discussion

We observed significantly elevated hippocampal miR-206 expression in pilocarpine-evoked TLE mice in our model system. Our stereotactic hippocampal delivery experiments further confirmed that miR-206 antagomir could effectively knock down endogenous hippocampal miR-206, whereas its negative control or the TrkB-selective inhibitor ANA-12 did not alter miR-206 abundance. These observations indicate the high specificity of miR-206 antagomir for miR-206 suppression and also rule out indirect modulation of miR-206 as the working mechanism for ANA-12.

Consistent with previous bioinformatic predictions and functional reports [20,34,35], our experimental findings support a regulatory link between miR-206 and BDNF-dependent signaling in the epileptic hippocampus. Several prior studies have reported that miR-206 targets BDNF to mediate pathological changes in multiple central nervous system disorders, including Alzheimer’s disease, depression and subarachnoid hemorrhage [26,27,28,29]. However, few studies have applied TrkB pharmacological blockade to verify whether miR-206 exerts biological effects through BDNF/TrkB in temporal lobe epilepsy. In the present study, we used ANA-12-mediated TrkB inhibition for rescue validation and demonstrated that the protective outcomes of the miR-206 antagomir are BDNF/TrkB-dependent in pilocarpine-induced TLE mice. In our TLE model, miR-206 inhibition increased the expression of BDNF and its receptor TrkB, accompanied by elevated levels of the synaptic-plasticity-related proteins SYP and PSD95. Given the well-documented roles of BDNF/TrkB signaling in neuronal survival, injury repair, and synaptic remodeling under epileptic conditions [18,26,36,37], these molecular alterations likely underlie the anti-seizure and neuroprotective phenotypes observed after miR-206 antagomir treatment. The reversal of these beneficial outcomes following pharmacological TrkB blockade with ANA-12 further demonstrates that the protective effects triggered by miR-206 suppression are functionally dependent on intact BDNF/TrkB pathway activation. Changes in SYP and PSD95 detected in our model may reflect improved synaptic structural integrity after seizure-associated brain damage, as both molecules are established pre- and postsynaptic markers whose abundance correlates with neural circuit reconstruction after neuronal insult [28,29,30,31].

ANA-12 is a selective antagonist that binds to the TrkB receptor. It suppresses the activation of its downstream signaling cascades. We found that miR-206 antagomir administration effectively restored BDNF levels and enhanced neuronal survival in epileptic mice. Accumulating evidence has confirmed that miR-206 can inhibit BDNF/TrkB pathway activation by negatively regulating BDNF transcription and translation. Inhibition of miR-206 expression via antagomir relieves the targeted silencing effect on BDNF. This process reactivates the TrkB downstream neuroprotective signaling cascades. This finding is consistent with previous results in neuronal injury and neurodegeneration models. Moreover, the neuroprotective and pro-survival effects mediated by miR-206 inhibition largely depend on intact activation of the BDNF/TrkB axis. Blockade of TrkB by ANA-12 significantly reverses the beneficial phenotypes induced by miR-206 antagomir intervention. This result further verifies the dominant regulatory mechanism of miR-206/BDNF/TrkB signaling in epileptic neuronal damage repair. Our results showed that miR-206 antagomir treatment significantly enhanced BDNF/TrkB pathway activation in TLE mice. It upregulated the expression of SYP and PSD95 and mitigated neuronal damage and loss. In contrast, pharmacological blockade of the BDNF/TrkB pathway with ANA-12 reversed the beneficial effects of miR-206 antagomir. It also weakened the neuroprotective capacity of miR-206 antagomir against epilepsy-induced neuronal deficits.

EEG monitoring revealed that miR-206 antagomir injection markedly prolonged seizure latency in pilocarpine-induced epileptic mice. It reduced the frequency of chronic spontaneous seizures and shortened seizure duration. These results demonstrate that miR-206 inhibition exerts a potent anti-epileptic effect. In contrast, pretreatment with ANA-12 attenuated the anti-epileptic efficacy of miR-206 antagomir. This finding suggests that the neuroprotective effects of miR-206 antagomir against epilepsy are partially dependent on BDNF/TrkB pathway activation.

Morris Water Maze assays further validated the favorable impacts of miR-206 antagomir on cognitive performance in epileptic mice. During the spatial navigation phase, mice receiving miR-206 antagomir exhibited distinctly shorter escape latency relative to untreated epileptic animals. This result indicates improved spatial learning ability. In the spatial probe test, these mice devoted more time to the target quadrant. This quadrant was where the platform had been placed beforehand. They also crossed the original platform area more frequently. This finding reflects enhanced spatial memory capacity.

Hippocampus-dependent spatial learning and memory strongly rely on intact BDNF/TrkB signaling. This signaling regulates hippocampal synaptic plasticity and neural circuit function. Epilepsy-induced BDNF downregulation impairs synaptic transmission and causes cognitive impairment. Restored BDNF/TrkB activation effectively rescues spatial memory deficits in epileptic models.

The cognitive improvement induced by miR-206 inhibition is mediated by the BDNF/TrkB signaling pathway. MiR-206 knockdown upregulates BDNF and activates TrkB signaling. This process enhances hippocampal synaptic plasticity and attenuates epilepsy-induced neuronal loss. Such hippocampal structural and functional repairs effectively improve memory in TLE mice. Notably, ANA-12 administration reversed the cognitive improvements induced by miR-206 antagomir. This outcome further reinforces the central contribution of the BDNF/TrkB signaling pathway. This pathway mediates the neuroprotective effects triggered by miR-206 suppression.

HE staining and Nissl staining demonstrated that mice treated with miR-206 antagomir showed more regularly shaped and tightly arranged hippocampal CA3 neurons. They also had a significantly higher number of surviving neurons compared with untreated epileptic mice. The hippocampal CA3 region is essential for memory processing. Neuronal disorder and loss in this region are hallmark pathologies of epileptic brain damage. Recurrent seizures trigger neuronal Nissl body damage. This process leads to a disorganized hippocampal structure and reduced neuronal viability. Mechanistically, miR-206 inhibition restores BDNF/TrkB signaling. This signaling preserves neuronal morphology and Nissl body integrity. It further alleviates epilepsy-associated hippocampal neuronal injury. These results suggest that miR-206 antagomir alleviates hippocampal neuronal damage induced by epileptic seizures. Moreover, ANA-12 treatment weakened the neuroprotective effects of miR-206 antagomir on hippocampal neurons. This finding confirms that the neuronal protective effect of miR-206 inhibition is also mediated through the BDNF/TrkB signaling pathway.

Our findings supplement the regulatory network of miR-206 in epilepsy. They support the potential involvement of the miR-206/BDNF/TrkB/synaptic plasticity axis. Pharmacological inhibition using ANA-12 further supports the TrkB-dependent mechanism of miR-206 action. It provides preliminary mechanistic evidence for its targeted application. Inhibiting miR-206 ameliorates epileptic manifestations and relieves epilepsy-related neuronal injury by regulating the BDNF/TrkB signaling pathway. Given its vital role in modulating neuronal survival and synaptic function, targeted suppression of miR-206 may be a potential adjuvant therapy for refractory epilepsy.

This work further clarifies the molecular mechanism of temporal lobe epilepsy. It provides a preliminary experimental basis and a potential target for miRNA-based clinical treatment of intractable epilepsy. We will systematically explore the precise targeting and regulatory relationship between miR-206 and BDNF in our future independent research. Additional regulatory mechanisms may also mediate the beneficial effects of miR-206 inhibition. Further studies are needed, including validation in large animal models and clinical translational investigations. These studies will clarify the underlying mechanisms and evaluate the clinical applicability of miR-206 antagomir for epilepsy treatment.

One important limitation of this study is that the normal control baseline miR-206 dataset was obtained from an independent cohort of experimental animals. These con-trol animals were processed with identical experimental workflows. All raw baseline values are provided in the Supplementary Materials of this article. A further statistical limitation should also be noted. We applied Fisher’s LSD for post hoc pairwise com-parison under equal variance conditions. This test does not adjust for multiple comparisons and is less conservative compared with Tukey’s HSD post hoc test. Re-analysis of our dataset indicates our main experimental conclusions would not change even when using the more stringent Tukey’s HSD method. We adopted 5 independent biological replicates for molecular and histological assays to improve normality assessment and SEM stability. 3 technical replicates were performed for each biological sample to reduce measurement variation. Technical replicates were not used as independent statistical units. Biological variation still exists within the pilocarpine-induced TLE mouse model, and further larger-scale animal studies are required to fully validate these findings.

## 5. Conclusions

Collectively, this study demonstrates that miR-206 is significantly upregulated in the hippocampus of pilocarpine-induced epileptic mice. miR-206 antagomir effectively downregulates miR-206 expression, enhances BDNF/TrkB signaling, upregulates synaptic plasticity-related proteins SYP and PSD95, reduces seizure frequency and duration, improves spatial learning and memory, and alleviates hippocampal neuronal damage. The TrkB antagonist ANA-12 reverses these beneficial effects, confirming that the antiepileptic and neuroprotective actions of miR-206 antagomir rely on functional BDNF/TrkB pathway activation. Our findings expand the regulatory network of miR-206 in epilepsy and support the miR-206/BDNF/TrkB-synaptic-plasticity regulatory axis, with ANA-12 pharmacological intervention further validating this TrkB-dependent mechanism. Distinct from conventional symptomatic antiepileptic agents, targeting miR-206 exerts combinatorial benefits including seizure suppression, neuronal injury repair, and cognitive improvement, which may offer a potential adjuvant therapeutic strategy for refractory temporal lobe epilepsy. Nevertheless, the precise targeting relationship between miR-206 and BDNF requires further independent investigation. Additional regulatory pathways may also contribute to the protective outcomes triggered by miR-206 inhibition. Future work, including large-animal studies and translational research, will be necessary to further characterize the mechanism and clinical applicability of miR-206 antagomir for epilepsy treatment.

## Data Availability

The original contributions presented in this study are included in the article/Supplementary Material. The normal control baseline dataset can be accessed within Supplementary Figure S1 and Supplementary Table S1. Further inquiries can be directed to the corresponding author.

## Supplementary Materials

The following supporting information can be downloaded at: https://www.mdpi.com/article/10.3390/brainsci16101048/s1, Table S1: Protein expression levels of BDNF. Figure S1: Protein expression levels of BDNF in the hippocampus of normal control mice and epileptic mice. (A) Quantitative analysis of relative protein expression levels normalized to GAPDH. (B) Representative Western blot bands of target proteins. Data are presented as mean ± SEM *n* = 5 independent biological replicates per group each replicate derived from one individual mouse with three technical replicate western blot runs for each sample. Statistical significance was determined by one-way ANOVA followed by the LSD test for homogeneous variances or Tamhane’s T2 test for heterogeneous variances. (ns *p* > 0.05, * *p* < 0.05, ** *p* < 0.01, *** *p* < 0.001).

## Abbreviations

The following abbreviations are used in this manuscript:

TLE	Temporal Lobe Epilepsy
PILO	Pilocarpine
AEDs	Anti-epileptic Drugs
MiRNA	MicroRNA
EEG	Electroencephalogram
SRS	Spontaneous Recurrent Seizures
CNS	Central Nervous System
BDNF	Brain-derived Neurotrophic Factor
TrkB	Tyrosine kinase receptor B
SYP	Synaptophysin
PSD95	Postsynaptic density protein 95
WB	Western blotting
HE	Hematoxylin–eosin

## Appendix A

**Table A1:** Relative miR-206 expression in the control group and TLE model group.

Groups	Relative miR-206 Level
Control	1.0 ± 0.04
TLE	2.5 ± 0.07

**Table A2:** Relative miR-206 expression in five groups.

Groups	Relative miR-206 Level
TLE	1.00 ± 0.03
TLE + miR-206AC	0.97 ± 0.01
TLE + miR-206A	0.32 ± 0.01
TLE + ANA12	1.02 ± 0.02
TLE + miR-206A + ANA12	0.35 ± 0.01

**Table A3:** SRS in epileptic mice evaluated by EEG.

Groups	SRS Latency (Day)	SRS Frequency (per Day)	Seizure Duration (s)
TLE	12.70 ± 0.62	4.20 ± 0.39	35.70 ± 1.45
TLE + miR-206 AC	11.30 ± 0.33	4.00 ± 0.58	35.00 ± 2.08
TLE + miR-206 A	16.60 ± 0.67	2.30 ± 0.33	24.30 ± 1.76
TLE + ANA-12	10.10 ± 0.58	5.60 ± 0.29	37.00 ± 1.16
TLE + miR-206 A + ANA-12	14.30 ± 0.86	3.60 ± 0.31	25.60 ± 1.45

**Table A4:** Escape latency in the Morris water maze place navigation test.

Groups	Training Day 1 (s)	Training Day 2 (s)	Training Day 3 (s)	Training Day 4 (s)
TLE	86.0 ± 2.3	82.0 ± 1.2	73.6 ± 1.4	66.0 ± 2.6
TLE + miR-206AC	84.4 ± 2.6	80.8 ± 2.9	75.0 ± 2.4	69.6 ± 1.7
TLE + miR-206A	69.0 ± 1.7	58.2 ± 2.4	52.8 ± 1.8	50.0 ± 1.6
TLE + ANA-12	88.6 ± 1.1	86.0 ± 2.3	78.0 ± 1.5	73.2 ± 2.3
TLE + miR-206A + ANA-12	81.6 ± 2.0	73.2 ± 1.2	68.6 ± 2.4	65.0 ± 1.7

**Table A5:** the spatial probe test in the Morris water maze.

Groups	Time Spent in the Platform Quadrant (s)	Number of Platform Crossings (Count)
TLE	36.4 ± 3.2	1.4 ± 0.50
TLE + miR-206AC	36.2 ± 4.3	1.6 ± 0.51
TLE + miR-206A	51.0 ± 3.4	4.8 ± 0.37
TLE + ANA-12	23.4 ± 2.0	1.2 ± 0.36
TLE + miR-206A + ANA12	42.8 ± 2.6	2.8 ± 0.37

**Table A6:** quantitative analysis of hippocampal CA3 neurons.

Groups	Hippocampal CA3 Neurons (Count)
TLE	80.33 ± 4.67
TLE + miR-206AC	81.33 ± 4.33
TLE + miR-206A	103.33 ± 3.48
TLE + ANA12	62.67 ± 2.33
TLE + miR-206A + ANA12	79.33 ± 2.45

**Table A7:** Protein expression levels of BDNF, TrkB, SYP, and PSD95.

Groups	BDNF Level	TrkB Level	SYP Level	PSD95 Level
TLE	1.00 ± 0.047	1.00 ± 0.026	1.00 ± 0.026	1.00 ± 0.034
TLE + miR-206AC	1.02 ± 0.014	0.95 ± 0.023	0.99 ± 0.032	0.94 ± 0.017
TLE + miR-206A	1.45 ± 0.038	1.21 ± 0.031	1.16 ± 0.031	1.19 ± 0.021
TLE + ANA-12	0.93 ± 0.046	0.81 ± 0.026	0.85 ± 0.038	0.83 ± 0.023
TLE + miR-206A + ANA-12	1.49 ± 0.016	1.05 ± 0.025	1.03 ± 0.023	1.07 ± 0.025
