# Supplementary material for: Inhibition of miR-206 Ameliorates Seizures and Cognitive Deficits via BDNF/TrkB Signaling in Pilocarpine-Induced TLE Mice
Source: Brain Sci. 2026 Sep 29;16(10):1048. doi: 10.3390/brainsci16101048 (PMC13702561; doi:10.3390/brainsci16101048)
Supplement: Supplementary file 1 [file brainsci-16-01048-s001.zip › brainsci-4552660-supplementary.pdf]

Table S1. Protein expression levels of BDNF.

Groups	BDNF Level
Normal control	1.00± 0.015
TLE	0.65 ± 0.03

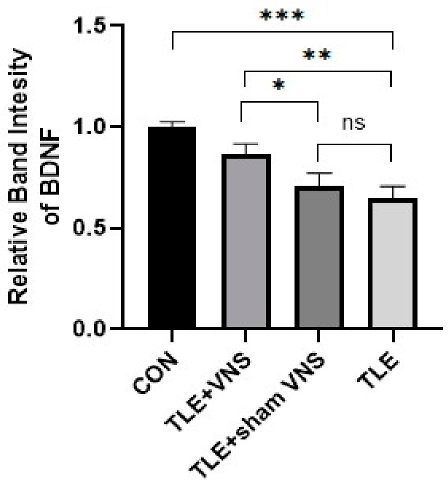

A

B BDNF

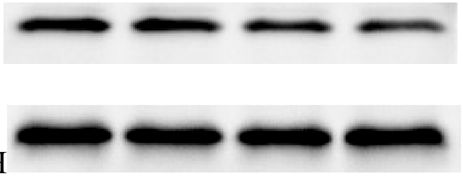

28KDa

GAPDH 36KDa

Normal CON TLE+VNS TLE+shan VNS TLE

Figure S1. Protein expression levels of BDNF in the hippocampus of normal control mice and epileptic mice. (A) Quantitative analysis of relative protein expression levels normalized to GAPDH. (B) Representative Western blot bands of target proteins. Data are presented as mean ± SEM n = 5 independent biological replicates per group each replicate derived from one individual mouse with three technical replicate western blot runs for each sample. Statistical significance was determined by one-way ANOVA followed by the LSD test for homogeneous variances or Tamhane’s T2 test for heterogeneous variances. (ns $p > 0.05$, * $p < 0.05$, ** $p < 0.01$, *** $p < 0.001$).
